# Exploring the Knowledge, Attitudes, and Perceptions of Hospital Staff and Patients on Environmental Sustainability in the Operating Room: Quality Improvement Survey Study

**DOI:** 10.2196/59790

**Published:** 2024-11-28

**Authors:** Nicole Kasia Stachura, Sukham K Brar, Jacob Davidson, Claire A Wilson, Celia Dann, Mike Apostol, John Vecchio, Shannon Bilodeau, Anna Gunz, Diana Catalina Casas-Lopez, Ruediger Noppens, Ken Leslie, Julie E Strychowsky

**Affiliations:** 1 Schulich School of Medicine and Dentistry Western University London, ON Canada; 2 Division of Paediatric Surgery Children’s Hospital London Health Sciences Centre London, ON Canada; 3 Department of Surgery Western University London Health Sciences Centre London, ON Canada; 4 Facilities Management London Health Sciences Centre London, ON Canada; 5 Department of Surgical Services London Health Sciences Centre London, ON Canada; 6 Department of Paediatrics Western University London, ON Canada; 7 Department of Anesthesia and Perioperative Medicine Western University London, ON Canada; 8 Department of Otolaryngology-Head and Neck Surgery Western University London Health Sciences Centre London, ON Canada

**Keywords:** environmental sustainability, sustainable healthcare, operating room, hospital, recycling, climate change, global warming, staff, patient, attitude, opinion, energy consumption

## Abstract

**Background:**

In Canada, the health care system has been estimated to generate 33 million metric tons of greenhouse gas emissions annually. Health care systems, specifically operating rooms (ORs), are significant contributors to greenhouse gas emissions, using 3 to 6 times more energy than the hospital’s average unit.

**Objective:**

This quality improvement study aimed to investigate the knowledge, attitudes, and perceptions of staff members and patients on sustainability in the OR, as well as identify opportunities for initiatives and barriers to implementation.

**Methods:**

A total of 2 surveys were developed, consisting of 27 questions for staff members and 22 questions for patients and caregivers. Topics included demographics, knowledge and attitudes regarding environmental sustainability, opportunities for initiatives, and perceived barriers. Multiple-choice, Likert-scale, and open-ended questions were used.

**Results:**

A total of 174 staff members and 37 patients participated. The majority (152/174, 88%) of staff members had received no and minimal training on sustainability, while 93% (162/174) cited practicing sustainability at work as moderately to extremely important. Among patients and caregivers, 54% (20/37) often or always noticed when a hospital is being eco-friendly. Both staff members and patients agreed that improving sustainability would boost satisfaction (125/174, 71.8% and 22/37, 59.4%, respectively) and hospital reputation (22/37, 59.4% and 25/37, 69.5%, respectively). The staff members’ highest-rated environmental initiatives included transitioning to reusables, education, and improved energy consumption, while patients prioritized increased nature, improved food sourcing, and education. Perceived barriers to these initiatives included cost, lack of education, and lack of incentives.

**Conclusions:**

Staff members and patients and caregivers in a large academic health care center acknowledge the significance of environmental sustainability in the OR. While they do not perceive a direct impact on patient care, they anticipate positive effects on satisfaction and hospital reputation. Aligning initiatives with staff members and patient and caregiver preferences can help drive meaningful change within the OR and beyond.

## Introduction

The World Health Organization labeled climate change as “the single biggest health threat facing humanity” [1]. In Canada, the health care system has been estimated to generate 33 million metric tons of greenhouse gas emissions annually [2,3]. Within the health care sector, operating rooms (ORs) are a significant portion of a hospital’s environmental footprint, using 3 to 6 times more energy than the hospital’s average unit [4-6]. Major sources of OR emissions stem from the reliance on single-use materials, biohazardous medical waste, and energy consumption [7,8]. Focusing on transforming the OR into a sustainable space presents a strategic opportunity to reduce the health care sector’s environmental footprint.

With this goal in mind, a multidisciplinary committee called the OR–Planetary Health Intervention Team (OR-PHIT) was created at the London Health Sciences Centre (LHSC) in London, Ontario, Canada. The team works to reengage the hospital in environmental initiatives and propose new ideas to reduce the environmental footprint of the OR. To drive effective change, the OR-PHIT must first understand the current perspectives of hospital staff members and patients. Despite some progress in related studies, the area remains relatively unexplored. A study conducted by the Department of Otolaryngology-Head and Neck Surgery at LHSC found that Canadian otolaryngologists strongly believe in climate change, but there was some ambivalence surrounding ORs being a strong contributor [9]. Other studies found varied barriers, such as lack of support from leadership and inadequate knowledge or education [10,11]. However, the mentioned studies were limited to specific departments and physicians.

As such, 2 surveys were created to characterize the knowledge, attitudes, and perceptions of hospital staff members, patients, and caregivers regarding environmental sustainability in the perioperative areas. This quality improvement initiative aimed to explore if improving the sustainability performance of the OR may impact workplace satisfaction and overall patient experience, aspects that have not been previously explored. It also aimed to identify opportunities for initiatives that will engage both staff members and patients while effectively reducing the OR’s environmental impact.

## Methods

### Survey Development

Separate surveys were developed for hospital staff members (ie, nurses, physicians, and OR aids), and patients and caregivers. For questionnaire development, multiple meetings were had to gain input from experts in the field. This included input from members of Western University Sustainability, the Child and Youth Advisory Council, and OR-PHIT. To help establish content validity, the qualifications of the individuals involved spanned from physicians and surgeons, surgical service staff members, and facilities management individuals at LHSC, all of whom have been involved in sustainability projects or research in the past. Once a questionnaire was developed, the surveys were pilot-tested on another small group from the OR-PHIT. The surveys consisted of 27 staff questions and 22 patient and caregiver questions. Questions were formatted using a combination of multiple choice, select all that apply, and a variety of Likert scales (full surveys can be referenced in Multimedia Appendix 1). They were distributed only to individuals in perioperative areas at all 4 hospital sites in London, Ontario, Canada. The surveys were launched on April 1, 2023, and remained open for 4 months for voluntary participation. No incentives were provided. Questions were displayed on 1 screen in a set order. Categories included demographics, knowledge, attitudes, opportunities, and barriers. There was a combination of multiple-choice, Likert-scale, and open-ended questions (see Multimedia Appendix 1 for full surveys), with participants able to skip and modify their answers before submission.

### Survey Dissemination

The survey was developed and administered anonymously using REDCap (Research Electronic Data Capture, Vanderbilt University) hosted at LHSC [12,13]. The surveys were promoted within the perioperative areas of each London hospital including Victoria (Children’s) Hospital, University Hospital, Nazem Kadri Surgical Centre, and St. Joseph’s Hospital. This was done using QR codes and advertisements at booths during hospital events such as Earth Week. As such, the population from which the sample was drawn included staff members working in the perioperative regions of these hospitals (ie, surgeons, nurses, anesthesiologists, managers, etc), as well as patients and their family members who have undergone surgery at one or more of these hospitals. A total of 211 participants completed any part of the survey. The sample size for the staff was 174, while the sample size for the patients and caregivers was 37.

### Data Analysis

Descriptive statistics, including median, IQR, and frequency of outcomes, were calculated. Differences between surgical and nonsurgical staff members and gender differences were explored using chi-square tests for categorical outcomes. An α level of .05 was used to determine statistical significance. All statistical analyses were completed using SAS software (version 9.4 SAS Institute Inc). All data including only partial responses were included in the analysis*.* For the open-ended questions, participant responses were analyzed by multiple research team members. Similar responses were grouped and common themes were determined.

### Ethical Considerations

The survey was part of a larger OR environmental sustainability project granted Ethics Board Exemption as a quality improvement project by the Western University Health Sciences Research Ethics Board (#121301). Participant data were kept confidential and anonymous through the REDCap hosted at Lawson Health Research Institute, a joint venture with LHSC. The participants were informed of the study purpose, estimated length, confidentiality, and intended use of data. Participation in the survey implied consent. The survey participants did not receive any form of compensation.

## Results

### Participant Characteristics

Out of 314 participants, 211 completed any part of the survey. This included a sample size of 174 staff members (73 surgical and 101 nonsurgical). The median age of staff members was 40 (IQR 33-51) years and the gender proportions were 74% (125/174) women, 21.3% (36/174) men, and 4.7% (8/174) preferred not to answer. The majority of staff members had worked at Victoria (Children’s) Hospital (134/174, 77%), followed by University Hospital (68/174, 39.1%), St. Joseph’s Hospital (18/174, 10.3%), and Nazem Kadri Surgical Centre (6/174, 3.5%). Of note, this question was formatted as “select all that apply”; therefore, the percentages will not equate to 100% for this question (Multimedia Appendix 1). Staff members had varied experience, with 29.1% (50/174) having <5 years, 16.9% (29/174) having 5-9 years, 27.9% (48/174) having 10-19 years, 17.4% (30/174) having 20-29 years, and 8.7% (15/174) having 30+ years.

The patient and caregiver sample size was 37, with 81.1% (30/37) women and 18.9% (7/37) men. Most have been patients at Victoria (Children’s) Hospital (29/37, 78.4%), followed by University Hospital (14/37, 37.8%), St. Joseph’s Hospital (7/37, 18.9%), and Nazem Kadri Surgical Centre (1/37, 2.7%). Of note, this question was formatted as “select all that apply”; therefore, the percentages will not equate to 100% (Multimedia Appendix 1). Most patients and caregivers had 4 or more surgeries at London hospitals (15/37, 40.5%), with 16.2% (6/37) having 3, a total of 18.9% (7/37) having 2, and 24.3% (9/37) having 1 surgery.

### Knowledge and Awareness

#### Staff Members

Approximately half of the staff members (89/174, 51.4%) had no (30/174, 17.3%) or minimal (59/174, 34.1%) knowledge about the causes of greenhouse gas emissions, whereas 26% (45/174) had “some,” 16.2% (28/174) had “moderate,” and 6.4% (11/174) had “strong” knowledge. In addition, more surgical staff members (43/74, 59%) rated having some, moderate, or strong knowledge compared with nonsurgical staff members (41/100, 41%; *P*=.01). Most staff members have received none (82/174, 47.4%) or limited (70/174, 40.5%) training regarding environmental sustainability in the workplace, whereas 6.4% (11/174) stated “minor,” 4.1% (7/174) stated “moderate,” and 1.7% (3/174) stated “plenty.” There were no significant differences between surgical staff members compared with nonsurgical staff members for the amount of training (*P*=.08). A total of 57.5% (100/174) of staff members stated that they were “very dissatisfied” or “dissatisfied” with the current sustainability performance of the operating room, whereas 37.4% (65/174) were “neutral,” and 5.2% (9/174) fell into the “satisfied” or “very satisfied” ranking. A total of 93.1% (162/174) of staff members indicated that practicing environmental sustainability at work was moderately (27/174, 15.5%), very (58/174, 33.3%), or extremely (77/174, 44.3%) important, whereas the other 7% (12/174) ranked “slightly” or “not” important. Finally, a total of 82.2% (143/174) strongly or somewhat agreed that they would like to learn more, whereas 10.3% (18/174) were neutral, and 7.5% (13/174) disagreed or strongly disagreed.

#### Patient

Over half (20/37, 54%) of patients and caregivers rated that they often (14/37, 37.8%) or always (6/37, 16.2%) notice when a hospital is environmentally friendly, whereas 29.7% (11/37) rated “sometimes,” 10.9% (4/37) rated “occasionally,” and 5.4% (2/37) rated “never.” A total of 47.3% (17/37) rated that they often (11/37, 30.6%) or always (6/37, 16.7%) think about how the hospital could improve its sustainability during their stay, whereas 11.1% (4/37) rated “sometimes,” 8.3% (3/37) rated “occasionally,” and 33.3% (12/37) rated “never.” Exactly half (18/37, 50%) of patients and caregivers have no (7/37, 19.4%) or minimal (11/37, 30.6%) knowledge of environmental sustainability in the perioperative areas, whereas 38.9% (14/37) have “some,” 11.1% (4/37) have “moderate,” and 0% (0/37) have “strong” knowledge. Nonetheless, 62.1% (23/37) strongly (12/37, 32.4%) or somewhat (11/37, 29.7%) agree that they would like to learn more, while 13.5% (5/37) were “neutral,” 10.8% (4/37) somewhat disagreed, and 13.6% (5/37) strongly disagreed.

### Attitudes and Perceptions

#### Staff Members

**Figure 1:**
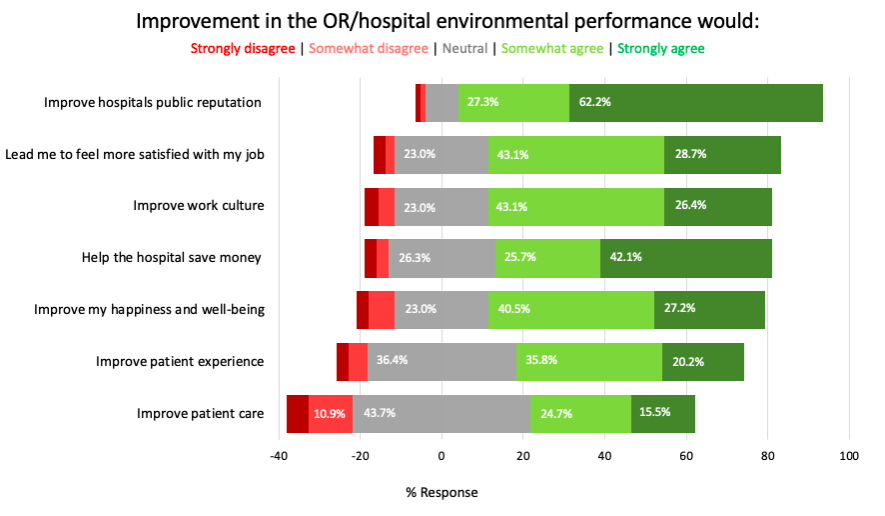
Attitudes and perceptions of staff members on the anticipated outcomes of improving operating room (OR) and hospital environmental sustainability performance.

Most staff members (149/174, 86.6%) consider the environment in daily decisions, and 89.4% (152/174) prioritize improving environmental sustainability. Most staff members strongly or somewhat agreed that improving sustainability would lead them to feel more satisfied with their job (125/174, 71.8%) and work culture (121/174, 69.5%; Figure 1). Fewer staff members somewhat or strongly agreed that it would lead to better patient care (70/174, 40.2%) or improve patient experience (97/174, 56%), which was significantly favored by the nonsurgical staff members (66/101, 65.4%) compared with surgical (31/73, 43.1%; *P=.*04). A higher proportion of staff members (154/174, 89.5%) somewhat or strongly agreed that improving sustainability would improve the hospital’s public reputation, with more nonsurgical staff members (94/101, 93.1%) agreeing compared with surgical staff members (60/73, 84.6%; *P*<.001). In addition, 67.8% (116/174) think it would help save the hospital money, with no significant differences between surgical and nonsurgical staff members (*P*=.11).

#### Patient

**Figure 2:**
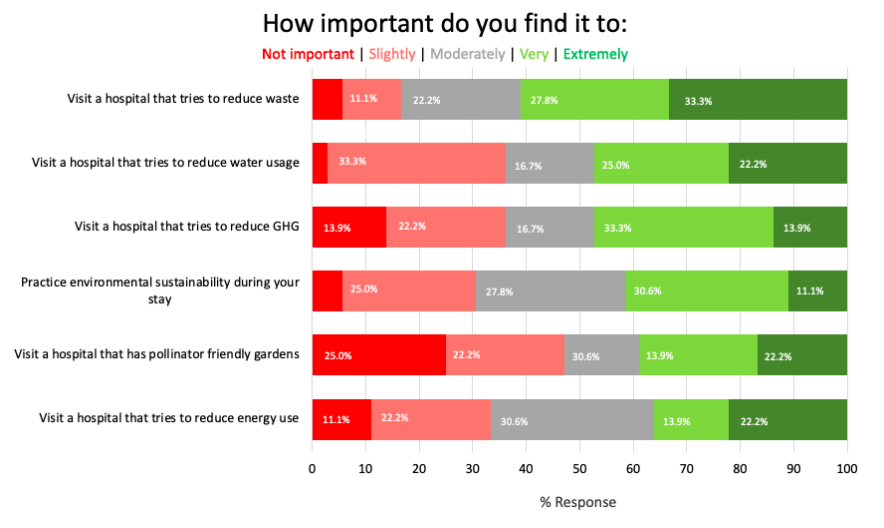
Patients’ perspectives on key values in hospital environmental practices. GHG: greenhouse gas.

Patients and caregivers somewhat or strongly agreed that environmental sustainability was important (29/37, 80.6%), considered it when making daily life decisions (29/37, 82.9%), and thought it should be a priority (28/37, 77.8%). More specifically, 69.5% (25/37) of patients and caregivers found it moderately to extremely important to practice environmental sustainability during their hospital stay. Of note, 83.3% (30/37) rated physical waste as moderately to extremely important, compared with 66.7% (25/37) for electricity usage, 63.9% (24/37) for water usage, and 52.8% (20/37) for pollinator-friendly gardens (Figure 2).

**Figure 3:**
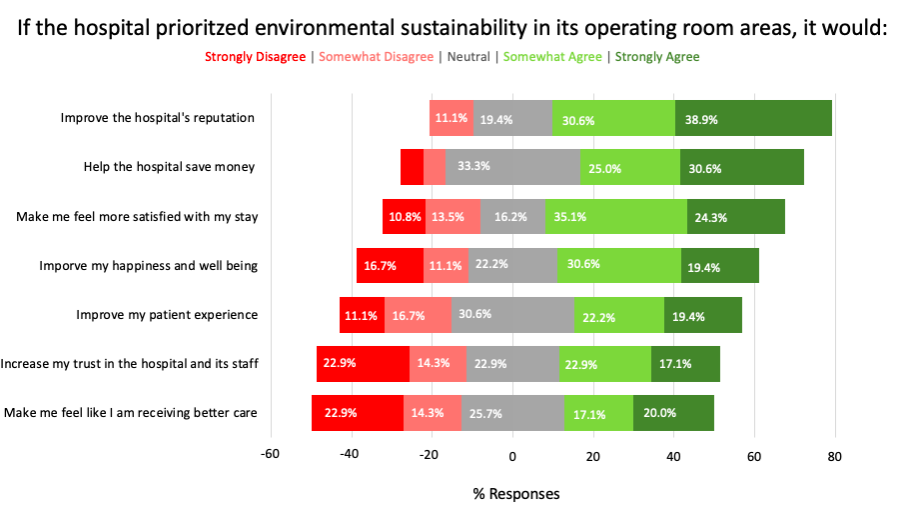
Attitudes and perceptions of patients on the anticipated outcomes of improving operating room and hospital environmental sustainability performance.

When asked how satisfied patients and caregivers were with the hospital’s current sustainability performance, most respondents were neutral (23/37, 62.2%), with 16.2% (6/37) stating they were dissatisfied or very dissatisfied. However, 59.4% (22/37) of patients and caregivers somewhat (13/37, 35.1%) or strongly (9/37, 24.3%) agreed that knowing the hospital prioritizes sustainability would make them feel more satisfied with their stay, and 50% (18/37) somewhat or strongly agreed that this would improve their happiness (Figure 3). In contrast, fewer staff members somewhat (6/35, 17.1%) or strongly (7/35, 20%) agreed that improved sustainability would make them feel like they are receiving better care. Furthermore, 40% (14/35) somewhat (8/35, 22.9%) or strongly (6/35, 17.1%) agreed it would increase their trust in hospital staff members. Finally, a total of 69.5% (25/36) of patients somewhat (11/36, 30.6%) or strongly (14/36, 38.9%) agreed that improving environmental sustainability would improve the hospital’s reputation, with 55.6% (20/36) somewhat (9/36, 25%) or strongly (11/36, 30.6%) agreeing that it would help the hospital save money.

### Opportunities and Barriers Identified by Staff Members and Patients

#### Staff Members

A full list of environmental opportunities rated by staff members can be found in Table 1. The highest-rated project was switching single-use items to reusable items (130/174, 74.7%). Education (85/174, 48.9%) and reducing the amount of energy used (69/174, 39.7%) were also highly rated, closely followed by reducing the amount of unused surgical instruments (68/174, 39.1%). To increase education, staff members would like to see an increase in email updates (97/174, 55.8%), posters or signage (95/174, 54.6%), and grand rounds or in-services (90/174, 51.7%). However, it should be noted that emails were significantly favored by the nonsurgical staff members group (*P*=.01), whereas grand rounds were favored by surgical staff members (*P*=.03).

A total of 11 surgical and 31 nonsurgical staff members participated in the open-ended portion, where they were invited to comment about alternative opportunities. Anticipated themes included those that are mentioned in Sergeant et al [14] discussing opportunities for greenhouse gas reductions and cost savings in hospitals. For surgical staff members, the most common themes identified were recycling, with 91% (10/11) of responses mentioning this domain, as well as education (4/11, 36%). For nonsurgical staff members, recycling was also the most common theme (12/31, 39%), as well as switching to reusable items (9/31, 29%) and decreasing transportation (6/31, 19%). Other themes included opportunities to improve education, food sourcing, or energy usage. The top 3 rated barriers identified by staff members were cost (115/174, 66.1%), lack of education (106/174, 60.9%), and lack of incentive (106/174, 60.9%).

**Table 1:** Opportunities rated by staff members for environmental sustainability initiatives.

Variables			Overall (n=174), n (%)		Surgical staff (n=73), n (%)		Nonsurgical staff (n=101), n (%)		*P* value
“**Please select the top 3 sustainability projects you think should be prioritized”**									
	Email updates	97 (55.8)		32 (43.8)		65 (64.4)		.01	
	Posters and signage	95 (54.6)		43 (58.9)		52 (51.5)		.33	
	Grand-rounds and in-services	90 (51.7)		45 (61.6)		45 (44.6)		.03	
	Learning modules	80 (46)		25 (34.3)		55 (54.5)		.01	
	Social media	60 (34.5)		20 (27.4)		40 (39.6)		.10	
	Other (please specify)	17 (9.8)		5 (6.9)		12 (11.9)		.27	
“**Please select the top 3 sustainability projects you think should be prioritized”**									
	Education and training of staff and patients	85 (48.9)		37 (50.7)		48 (47.5)		.69	
	Reduce the amount of energy used (ie, lighting, heating, cooling)	69 (39.7)		23 (31.5)		46 (45.5)		.06	
	Reducing the amount of unused surgical instruments (ie, less unnecessary sterilization)	68 (39.1)		44 (60.3)		24 (23.8)		<.001	
	Optimizing drugs and devices (ie, switching to low-carbon anesthetic gas)	59 (33.9)		29 (39.7)		30 (29.7)		.17	
	Better labeling of products and waste bins	57 (32.8)		22 (30.1)		35 (34.7)		.53	
	Optimizing food sourcing (eg, improving patient-provided food sourcing, plant-based foods)	43 (24.7)		9 (12.3)		34 (33.7)		.001	
	Increasing exposure to nature (eg, Nature for Healing)	39 (22.4)		8 (11)		31 (30.7)		<.01	
	Increasing leadership to create a culture of sustainability and meet goals	31 (17.8)		14 (19.2)		17 (16.8)		.69	
	Switching from single-use items to reusable items (eg, plastic garment bags, surgical gowns, and caps)	13 (74.7)		57 (78.1)		73 (72.3)		.38	

#### Patients

Several opportunities were rated by patients and caregivers (Table 2). The top-rated initiatives were increasing exposure to nature (22/37, 59.5%), improving food sourcing (21/37, 56.8%), education (20/37, 54.1%), and better waste labeling (20/37, 54.1%). In addition, 50% (20/40) of patients and caregivers would like to get involved in initiatives that improve environmental sustainability*.* A total of 5 patients participated in the open-ended section, with the main theme being improved food sourcing (3/5, 60%).

**Table 2:** Opportunities rated by patients for environmental sustainability initiatives.

Variables			Overall (n=37), n (%)		Nonsurgical patient (n=5), n (%)		Surgical patient (n=32), n (%)
“**Please select sustainability initiatives you would like to see during your hospital stay”**							
	Increased exposure to nature (eg, Nature for Healing)	22 (59.5)		4 (80)		18 (56.3)	
	Improved food sourcing (eg, patient-improved food sourcing, plant-based foods)	21 (56.8)		4 (80)		18 (56.3)	
	Education and training of staff members and patients	20 (54.1)		2 (40)		18 (56.3)	
	Better labeling of products and waste bins	20 (54.1)		2 (40)		18 (56.3)	
	Switching from single-use items to reusable items (eg, plastic garment bags, surgical gowns, and caps)	19 (51.4)		3 (60)		16 (50)	
	Reducing the amount of unused surgical instruments (ie, less unnecessary sterilization)	18 (48.7)		3 (60)		15 (46.9)	
	Reduce the amount of energy used (ie, lighting, heating, cooling)	11 (29.7)		1 (20)		10 (31.3)	
	Optimizing drugs and devices (ie, switching to low-carbon anesthetic gas)	11 (29.7)		2 (40)		9 (28.1)	

## Discussion

### Perspectives and Opportunities Identified by Staff Members

Survey findings reveal knowledge gaps on greenhouse gases and environmental projects, paralleling perioperative staff member experiences in the literature [9-11,15,16]. Limited workplace education contributes to this, but staff members express eagerness for more training [15,16]. Dissatisfaction with the current sustainability performance within the OR is evident, with beliefs that enhancing sustainability can boost job satisfaction and influence work culture. While fewer believe environmental efforts will impact patient care, a larger proportion perceive benefits in the hospital’s reputation, sustainability, and cost savings.

Staff members propose transitioning to reusable items, which has the potential to reduce the carbon footprint by 38% to 50% [17-19]. Switching to reusable gowns and masks offers a promising reduction in energy use and waste production [20,21]. Replacing disposable plastic for instrument trays with reusable alternatives and minimizing medical product packaging are suggested. Education initiatives are also crucial, with preferences for email updates, posters, and grand rounds. Staff members proposed “lunch and learns” and a “Green Team Newsletter'” in the open-ended section. There is also potential for leveraging social media and learning modules, although they ranked lower. Incorporating modules early in medical and nursing education has previously been shown to be successful [22]. Finally, reducing energy consumption is prioritized, given the OR’s higher energy intensity [4]. A previous study done in 30 ORs in North Carolina showed that turning off all anesthesia and OR equipment not in use saved 234.3 metric tons of CO_2_ emission per year and US $33,004 annually [23].

Additional initiatives include optimizing drugs and devices, such as adopting low-carbon anesthetic gases. A single OR anesthetist’s daily routine can emit the equivalent of “driving over 1000 km per day” depending on the chosen volatile agent for balanced general anesthesia [24]. The OR-PHIT has used educational efforts to decrease desflurane use by 24.5% across London hospitals in 2 years, cutting 473 metric tons of CO_2_, equivalent to ~2.3 million km driven by car [25]. Improving recycling and waste bin labeling is another opportunity as 90% of nonclinical waste is misclassified as hazardous waste and 50% of materials in sharps containers are nonsharps [26-28]. Strategies like increasing bins, designing signs, and educating staff members can result in cost savings [29]. Staff members suggest waste management process tours, with 1 stating, “We had a great in-service recently about waste management, and I would love to see it further expanded.”

### Perspectives and Opportunities Identified by Patients

This study sheds light on previously unexplored patient perspectives on environmental sustainability in the perioperative setting. Most patients have minimal knowledge about environmental sustainability but express interest in learning more. A significant percentage regularly notice when a hospital makes efforts to be environmentally friendly. Patients emphasize the importance of hospitals actively measuring and reducing greenhouse gas emissions, with a focus on waste reduction. Only a minority express satisfaction with the environmental performance of hospitals they visit.

The top opportunity identified by patients was exposure to nature, suggesting strategies like mimicking natural environments and enhancing access to outdoor gardens [30,31]. A program at LHSC, Nature for Healing, works on increasing patient and family experience through nature exposure [32]. Patients also prioritize improving food sourcing and reducing waste. Indeed, ~50% of patients leave most of their meals uneaten while in hospital [33]. In our survey, patients commented, “Hospital food waste troubles me a lot,” and suggested, “changing food suppliers to decrease waste of food and [single use] containers would increase patient satisfaction.” Existing initiatives have redesigned menus to be healthier and created local gardens for patient use [34,35]. Finally, educational initiatives through pamphlets, posters, and social media, as well as avenues for feedback can increase awareness. Allowing avenues for feedback and suggestions can also foster a sense of involvement and ownership while increasing awareness.

### Perceived Barriers

The top 3 staff member–identified barriers included cost, lack of education, and lack of incentive. Cost concerns involve expenses for new infrastructure, equipment, and staff members’ training [36]. However, certain initiatives, like reducing desflurane gas use, can yield potential savings [14]. Despite higher upfront costs for sustainability initiatives, such as reusable gowns, long-term savings make them cost-effective [37]. Lack of incentives and education were also identified barriers, by a systematic review of environmental sustainability in the OR [38]. Educational initiatives, such as an in-service tour, can enhance staff members’ understanding. One respondent suggested, “If staff members knew recycling and being sustainable can lower our costs, they may be more incentivized to help out.”

### Moving Forward

This survey reveals staff members’ and patient views on environmental sustainability opportunities and barriers in the OR. An intriguing question arises: do highly rated initiatives align with those proven to have the most substantial impact on reducing greenhouse gas emissions? Sergeant et al [14] use a peach tree diagram to compare the impact of interventions on greenhouse gas emissions and costs across 7 different categories [36]. While effective interventions like low-carbon buildings tend to be costly, others, such as desflurane reduction, achieve significant greenhouse gas emission reduction with annual savings [39]. Optimizing plant-rich diets, adding an energy manager, and switching to reusable gowns offer lower-cost carbon emission reductions. Hospitals prioritizing sustainability should evaluate effectiveness, costs, and savings when choosing initiatives. Considering staff members’ and patient perspectives is crucial, given their significant role. Hospital leadership can use this information with existing action guidelines to make decisions about reducing their carbon footprint [39].

### Limitations

Self-selection bias may exist as participants voluntarily chose to participate in the study, potentially skewing the sample toward those more interested in environmental sustainability. Challenges in estimating the total OR staff members limited the ability to accurately calculate the response rate. Limited patient and caregiver participation resulted in a small sample size. Since we used the anonymous public survey in REDCap, IP addresses were not captured; therefore, we did not have the capacity to determine if each participant was unique. Finally, the study exclusively captures perspectives from London, Ontario hospitals, potentially limiting generalizability.

### Conclusion

This quality improvement study explores patient and provider perspectives on environmental sustainability in perioperative areas. It reveals that while sustainability is not perceived to impact patient care directly, the participants anticipate positive effects on sustainability performance, staff members and patient satisfaction, and hospital reputation. We also identified opportunities and barriers to inform decision-making on initiatives aimed at reducing the hospital’s environmental impact.

## Appendix

Multimedia Appendix 1 Full staff/patient survey: environmental sustainability in the hospital and operating room.

## Abbreviations

LHSC: London Health Sciences Centre
OR-PHIT: Operating Room–Planetary Health Intervention Team
OR: operating room
REDCap: Research Electronic Data Capture
