# Supplementary material for: Exploring the Knowledge, Attitudes, and Perceptions of Hospital Staff and Patients on Environmental Sustainability in the Operating Room: Quality Improvement Survey Study
Source: JMIR Perioper Med. 2024 Nov 28;7:e59790. doi: 10.2196/59790 (PMC11638685; doi:10.2196/59790)
Supplement: Multimedia Appendix 1 [file periop_v7i1e59790_app1.pdf]

Environmental Sustainability in the Hospital and Operating Room

Purpose of this Quality Improvement Project:

The purpose of this study is to examine the personal beliefs and attitudes of hospital patients and staff surrounding environmental sustainability in the hospital and operating room.

Estimated Length of Survey:

Approximately 10 minutes.

Confidentiality:

All data collected from this survey is confidential and will remain anonymous. No individual data will be reported, aside from responses to open-ended questions. If any open-ended questions contain identifiable information, the statements will be adjusted to omit the identifiable information. You will be able to close and cancel participation in the survey at any time.

Intended use of Data:

This data will be published in a peer-reviewed journal and presented at medical conferences. The data will be stored and retained for 15 years following study cessation, after which point it will be destroyed and electronic database dismantled.

Please click 'Submit' to continue.

I am a...	◯ Patient/visitor/caregiver<br>◯ Staff member
I, or the person I am visiting, received surgical care at a London hospital:	◯ Yes<br>◯ No
I am a...	◯ Surgical staff<br>◯ Non-surgical staff

Please click "Submit" to continue.

DEMOGRAPHICS

I am a...

- ◯ Patient
- ◯ A family member/caregiver/friend/visitor of the patient

How old are you?

____

What is your gender identity?

- ◯ Man
- ◯ Woman
- ◯ Non-binary
- ◯ Another gender not listed
- ◯ Prefer not to say

I, or the person I am visiting, received surgical care at the following hospital(s).

- ☐ Children's Hospital, London Health Sciences Centre
- ☐ Victoria Hospital, London Health Sciences Centre
- ☐ University Hospital, London Health Sciences Centre
- ☐ Nazem Kadri Surgical Centre, London Health Sciences Centre
- ☐ St. Joseph's Health Care London

(Please select all that apply)

How many total times have you, or the person you are accompanying, had surgery in a London hospital?

- ◯ 1
- ◯ 2
- ◯ 3
- ◯ 4+

KNOWLEDGE/AWARENESS

Please state how often each one applies to you:

	Never	Occasionally	Sometimes	Often	Always
Environmental sustainability is something I look out for when I visit the hospital	◯	◯	◯	◯	◯
I notice when a hospital tries to be environmentally friendly	◯	◯	◯	◯	◯
I think about ways the hospital could improve its environmental sustainability during my visit(s)	◯	◯	◯	◯	◯

How would you describe your current knowledge regarding environmental sustainability in the hospital/operating room (including waste production and management, use of drugs and devices, energy usage etc.)

- ◯ No knowledge
- ◯ Minimal knowledge
- ◯ Some knowledge
- ◯ Moderate knowledge
- ◯ Strong knowledge

Please rate your level of agreement with the following statement:
I would like to learn more about environmental sustainability in the hospital/operating room

◯ Strongly disagree
◯ Somewhat disagree
◯ Neutral
◯ Somewhat agree
◯ Strongly agree

ATTITUDES/PERCEPTIONS

How important are each of the following to you?

	Not important	Slightly important	Moderately important	Very important	Extremely important
Practicing environmental sustainability during your hospital stay or visit	◯	◯	◯	◯	◯
Visiting a hospital that measures and tries to reduce its greenhouse gas emissions	◯	◯	◯	◯	◯
Visiting a hospital that measures and tries to reduce its waste	◯	◯	◯	◯	◯
Visiting a hospital that measures and tries to reduce its water usage	◯	◯	◯	◯	◯
Visiting a hospital that measures and tries to reduce its electricity/energy	◯	◯	◯	◯	◯
Visiting a hospital that has pollinator friendly gardens	◯	◯	◯	◯	◯

How satisfied are you with the hospital's current sustainability performance during your visits?

◯ Very dissatisfied
◯ Dissatisfied
◯ Neutral
◯ Satisfied
◯ Very satisfied

Please state your level of agreement or disagreement for the following statements.

Knowing that the hospital/operating room prioritizes environmental sustainability would:

	Strongly disagree	Somewhat disagree	Neutral	Somewhat agree	Strongly agree
Make me feel more satisfied with my hospital visit	◯	◯	◯	◯	◯

Improve my happiness and well-being during my stay	◯	◯	◯	◯	◯
Make me feel like I am receiving better care	◯	◯	◯	◯	◯
Increase my trust in the hospital and its staff	◯	◯	◯	◯	◯

Please state your level of agreement or disagreement for the following statements.

Improving the hospitals/operating rooms environmental sustainability would:

	Strongly disagree	Somewhat disagree	Neutral	Somewhat agree	Strongly agree
Be a valuable contribution to mitigating global climate change	◯	◯	◯	◯	◯
Improve patient experience	◯	◯	◯	◯	◯
Improve the hospital's public reputation	◯	◯	◯	◯	◯
Help the hospital save money	◯	◯	◯	◯	◯

OPPORTUNITIES FOR INVOLVEMENT/IMPROVEMENT INITIATIVES

Please select sustainability initiatives you would like to see during your hospital visits:
(Please select all that apply)

- ☐ Education/training of staff and patients
- ☐ Switching single-use items to reusable items (ex. plastic garment bags, surgical gowns/caps)
- ☐ Increasing exposure to nature (ex. Nature for Healing)
- ☐ Optimizing drugs and devices (ex. Switching to low carbon anaesthetic gases)
- ☐ Improved food sourcing (ex. patient provided food sourcing, plant-based foods)
- ☐ Reducing the amount of unused surgical instruments (i.e., less unnecessary sterilization)
- ☐ Reducing the amount of energy used (i.e., lighting, heating/cooling)
- ☐ Better labelling of products and waste bins

Are there any other opportunities for improvement not listed that you can identify?

Please state the level of agreement with the following statements.

	Strongly disagree	Somewhat disagree	Neutral	Somewhat agree	Strongly agree
In general, environmental sustainability is important to me	◯	◯	◯	◯	◯
I consider the environment when making decisions in my daily life	◯	◯	◯	◯	◯

I believe that improving environmental sustainability should be a priority	○	○	○	○	○
I would like to get involved in initiatives that help improve environmental sustainability	○	○	○	○	○

Any other comments you would like to add?

DEMOGRAPHICS

I am a...

- ◯ Patient
- ◯ A family member/caregiver/friend/visitor of the patient

How old are you?

____

What is your gender identity?

- ◯ Man
- ◯ Woman
- ◯ Non-binary
- ◯ Another gender not listed
- ◯ Prefer not to say

I, or the person I am visiting, received care at the following hospital(s).

- ☐ Children's Hospital, London Health Sciences Centre
- ☐ Victoria Hospital, London Health Sciences Centre
- ☐ University Hospital, London Health Sciences Centre
- ☐ Nazem Kadri Surgical Centre, London Health Sciences Centre
- ☐ St. Joseph's Health Care London

(Please select all that apply)

How many total times have you, or the person you are accompanying, visited a London hospital?

- ◯ 1
- ◯ 2
- ◯ 3
- ◯ 4+

KNOWLEDGE/AWARENESS

Please state how often each one applies to you:

	Never	Occasionally	Sometimes	Often	Always
Environmental sustainability is something I look out for when I visit the hospital	◯	◯	◯	◯	◯
I notice when a hospital tries to be environmentally friendly	◯	◯	◯	◯	◯
I think about ways the hospital could improve its environmental sustainability during my visit(s)	◯	◯	◯	◯	◯

How would you describe your current knowledge regarding environmental sustainability in the hospital (including waste production and management, use of drugs and devices, energy usage etc.)

- ◯ No knowledge
- ◯ Minimal knowledge
- ◯ Some knowledge
- ◯ Moderate knowledge
- ◯ Strong knowledge

Please rate your level of agreement with the following statement:
I would like to learn more about environmental sustainability in the hospital

◯ Strongly disagree
◯ Somewhat disagree
◯ Neutral
◯ Somewhat agree
◯ Strongly agree

ATTITUDES/PERCEPTIONS

How important are each of the following to you?

	Not important	Slightly important	Moderately important	Very important	Extremely important
Practicing environmental sustainability during your hospital stay or visit	◯	◯	◯	◯	◯
Visiting a hospital that measures and tries to reduce its greenhouse gas emissions	◯	◯	◯	◯	◯
Visiting a hospital that measures and tries to reduce its waste	◯	◯	◯	◯	◯
Visiting a hospital that measures and tries to reduce its water usage	◯	◯	◯	◯	◯
Visiting a hospital that measures and tries to reduce its electricity/energy	◯	◯	◯	◯	◯
Visiting a hospital that has pollinator friendly gardens	◯	◯	◯	◯	◯

How satisfied are you with the hospital's current sustainability performance during your visits?

◯ Very dissatisfied
◯ Dissatisfied
◯ Neutral
◯ Satisfied
◯ Very satisfied

Please state your level of agreement or disagreement for the following statements.

Knowing that the hospital prioritizes environmental sustainability would:

	Strongly disagree	Somewhat disagree	Neutral	Somewhat agree	Strongly agree
Make me feel more satisfied with my hospital visit	◯	◯	◯	◯	◯

Improve my happiness and well-being during my stay	○	○	○	○	○
Make me feel like I am receiving better care	○	○	○	○	○
Increase my trust in the hospital and its staff	○	○	○	○	○

Please state your level of agreement or disagreement for the following statements.

Improving the hospital's environmental sustainability would:

	Strongly disagree	Somewhat disagree	Neutral	Somewhat agree	Strongly agree
Be a valuable contribution to mitigating global climate change	○	○	○	○	○
Improve patient experience	○	○	○	○	○
Improve the hospital's public reputation	○	○	○	○	○
Help the hospital save money	○	○	○	○	○

OPPORTUNITIES FOR INVOLVEMENT/IMPROVEMENT INITIATIVES

Please select sustainability initiatives you would like to see during your hospital stay:
(Please select all that apply)

- ☐ Education/training of staff and patients
- ☐ Switching single-use items to reusable items (ex. plastic garment bags, surgical gowns/caps)
- ☐ Increasing exposure to nature (ex. Nature for Healing)
- ☐ Optimizing drugs and devices (ex. Switching to low carbon anaesthetic gases)
- ☐ Improved food sourcing (ex. patient provided food sourcing, plant-based foods)
- ☐ Reducing the amount of unused surgical instruments (i.e., less unnecessary sterilization)
- ☐ Reducing the amount of energy used (i.e., lighting, heating/cooling)
- ☐ Better labelling of products and waste bins

Are there any other opportunities for improvement not listed that you can identify?

Please state the level of agreement with the following statements.

	Strongly disagree	Somewhat disagree	Neutral	Somewhat agree	Strongly agree
In general, environmental sustainability is important to me	○	○	○	○	○
I consider the environment when making decisions in my daily life	○	○	○	○	○

I believe that improving environmental sustainability should be a priority	○	○	○	○	○
I would like to get involved in initiatives that help improve environmental sustainability	○	○	○	○	○

Any other comments you would like to add?

DEMOGRAPHICS	
What is your position/role?	◯ Anaesthesiology<br>◯ Surgery<br>◯ Nurse<br>◯ OR Aide<br>◯ Leader/Manager (please specify) ______<br>◯ Other (please specify) ______
Please select your position:	◯ Staff<br>◯ Fellow<br>◯ Resident<br>◯ Assistant
Please select your position:	◯ Staff<br>◯ Fellow<br>◯ Resident
Please select your location:	◯ OR<br>◯ Day Surgery<br>◯ PACU
How old are you?	______________________________
What is your gender identity?	◯ Man<br>◯ Woman<br>◯ Non-binary<br>◯ Another gender not listed<br>◯ Prefer not to say
What hospital(s) do you work at the most?	☐ Children's Hospital, London Health Sciences Centre<br>☐ Victoria Hospital, London Health Sciences Centre<br>☐ University Hospital, London Health Sciences Centre<br>☐ Nazem Kadri Surgical Centre, London Health Sciences Centre<br>☐ St. Joseph's Health Care London<br>(Please select all that apply)
How many years have you worked in the operating room or perioperative areas?	◯ < 5 years<br>◯ 5-9 years<br>◯ 10-19 years<br>◯ 20-29 years<br>◯ 30+ years

KNOWLEDGE

How would you rate your knowledge about causes of greenhouse gas emissions in the operating room (including waste production and management, use of drugs and devices, energy usage etc.)?

◯ No knowledge
◯ Minimal knowledge
◯ Some knowledge
◯ Moderate knowledge
◯ Strong knowledge

How would you rate your knowledge of environmental sustainability projects ongoing at the hospital?

◯ No knowledge
◯ Minimal knowledge
◯ Some knowledge
◯ Moderate knowledge
◯ Strong knowledge

How much training/education have you received regarding environmental sustainability in the operating rooms?

◯ None
◯ Limited
◯ Minor
◯ Moderate
◯ Plenty

Please rate your level of agreement with the following statement:
I would like to learn more about environmental sustainability in the hospital/operating room.

◯ Strongly disagree
◯ Somewhat disagree
◯ Neutral
◯ Somewhat agree
◯ Strongly agree

ATTITUDES/PERCEPTIONS

How important is practicing environmental sustainability at work to you?

◯ Not important
◯ Slightly important
◯ Moderately important
◯ Very important
◯ Extremely important

How satisfied are you with the current sustainability performance of the operating rooms you work in?

◯ Very dissatisfied
◯ Dissatisfied
◯ Neutral
◯ Satisfied
◯ Very satisfied

Please state the level of agreement for the following statements:

Improvement in the hospital/operating rooms environmental performance would:

	Strongly disagree	Somewhat disagree	Neutral	Somewhat agree	Strongly agree
Lead me to feel more satisfied with my job	○	○	○	○	○
Improve my happiness and well-being	○	○	○	○	○
Improve the work culture of the operating room	○	○	○	○	○
Help me to provide better patient care	○	○	○	○	○

Please state the level of agreement for the following statements:

Improving the hospitals/operating rooms environmental sustainability would:

	Strongly disagree	Somewhat disagree	Neutral	Somewhat agree	Strongly agree
Improve patient experience	○	○	○	○	○
Improve the hospital's public reputation	○	○	○	○	○
Help the hospital save money	○	○	○	○	○

OPPORTUNITIES FOR INVOLVEMENT/IMPROVEMENT INTIATIVES

Which of the following would you like to see to help increase education/awareness of environmental sustainability efforts?
(Please select all that apply)

☐ Posters/signage
☐ Learning modules
☐ Social media
☐ E-mail updates
☐ Grand rounds/in-services
☐ Other (please specify) ______

Please select 3 of the following sustainability projects that you think should be most prioritized:
(Please select 3)

☐ Education/training of staff and patients
☐ Switching single use items to reusable items (ex. plastic garment bags, surgical gowns/caps)
☐ Increasing exposure to nature (ex. Nature for Healing)
☐ Optimizing drugs and devices (ex. switching to low carbon anaesthetic gases)
☐ Optimizing food sourcing (ex. improving patient provided food sourcing, plant-based foods)
☐ Reducing the amount of unused surgical instruments (i.e., less unnecessary sterilization)
☐ Reducing the amount of energy used (i.e., lighting, heating/cooling)
☐ Increasing leadership to create a culture of sustainability and meet goals
☐ Better labelling of products and waste bins

Are there any opportunities for improvement not mentioned above that you can identify?

BARRIERS

Which of the following do you believe are barriers to improving the environmental sustainability in the operating room?
(Please select all that apply)

☐ Cost
☐ Lack of support
☐ Lack of education
☐ Lack of resources
☐ Negative attitudes
☐ Lack of time
☐ Lack of incentive
☐ Inconvenience

GENERAL KNOWLEDGE/ATTITUDES/PERCEPTIONS ON CLIMATE CHANGE

Please state the level of agreement with the following statements:

	Strongly disagree	Somewhat disagree	Neutral	Somewhat agree	Strongly agree
In general, environmental sustainability is important to me	○	○	○	○	○
I consider the environment when making decisions in my daily life	○	○	○	○	○
I believe that improving environmental sustainability should be a priority	○	○	○	○	○
I would like to get involved in initiatives that help improve environmental sustainability	○	○	○	○	○

Any other comments you would like to add?

DEMOGRAPHICS

What is your position/role?

- ◯ Physician/Trainee
- ◯ Nurse (RPN/RN)
- ◯ PSW
- ◯ Aide
- ◯ Administration professional/Clerk
- ◯ Porter
- ◯ Management & Leadership
- ◯ Housekeeping
- ◯ Security
- ◯ Therapist (OT, PT, SLP, RT, etc.)
- ◯ Other hospital staff (please specify) ______

Please specify your department:

____

How old are you?

____

What is your gender identity?

- ◯ Man
- ◯ Woman
- ◯ Non-binary
- ◯ Another gender not listed
- ◯ Prefer not to say

What hospital(s) do you work at the most?

- ☐ Children's Hospital, London Health Sciences Centre
- ☐ Victoria Hospital, London Health Sciences Centre
- ☐ University Hospital, London Health Sciences Centre
- ☐ Nazem Kadri Surgical Centre, London Health Sciences Centre
- ☐ St. Joseph's Health Care London

(Please select all that apply)

How many years have you worked at LHSC?

- ◯ < 5 years
- ◯ 5-9 years
- ◯ 10-19 years
- ◯ 20-29 years
- ◯ 30+ years

KNOWLEDGE

How would you rate your knowledge about causes of greenhouse gas emissions in the hospital (including waste production and management, use of drugs and devices, energy usage etc.)?

- ◯ No knowledge
- ◯ Minimal knowledge
- ◯ Some knowledge
- ◯ Moderate knowledge
- ◯ Strong knowledge

How would you rate your knowledge of environmental sustainability projects ongoing at the hospital?

◯ No knowledge
◯ Minimal knowledge
◯ Some knowledge
◯ Moderate knowledge
◯ Strong knowledge

How much training/education have you received regarding environmental sustainability in the hospital?

◯ None
◯ Limited
◯ Minor
◯ Moderate
◯ Plenty

Please rate your level of agreement with the following statement:
I would like to learn more about environmental sustainability in the hospital.

◯ Strongly disagree
◯ Somewhat disagree
◯ Neutral
◯ Somewhat agree
◯ Strongly agree

ATTITUDES/PERCEPTIONS

How important is practicing environmental sustainability at work to you?

◯ Not important
◯ Slightly important
◯ Moderately important
◯ Very important
◯ Extremely important

How satisfied are you with the current sustainability performance of the hospital you work in?

◯ Very dissatisfied
◯ Dissatisfied
◯ Neutral
◯ Satisfied
◯ Very satisfied

Please state the level of agreement for the following statements:

Improvement in the hospital environmental performance would:

	Strongly disagree	Somewhat disagree	Neutral	Somewhat agree	Strongly agree
Lead me to feel more satisfied with my job	◯	◯	◯	◯	◯
Improve my happiness and well-being	◯	◯	◯	◯	◯

Improve the work culture of the hospital	○	○	○	○	○
Help me to provide better patient care	○	○	○	○	○

Please state the level of agreement for the following statements:

Improving the hospital's environmental sustainability would:

	Strongly disagree	Somewhat disagree	Neutral	Somewhat agree	Strongly agree
Improve patient experience	○	○	○	○	○
Improve the hospital's public reputation	○	○	○	○	○
Help the hospital save money	○	○	○	○	○

OPPORTUNITIES FOR INVOLVEMENT/IMPROVEMENT INTIATIVES

Which of the following would you like to see to help increase education/awareness of environmental sustainability efforts?
(Please select all that apply)

☐ Posters/signage
☐ Learning modules
☐ Social media
☐ E-mail updates
☐ Grand rounds/in-services
☐ Other (please specify) ______

Please select 3 of the following sustainability projects that you think should be most prioritized:
(Please select 3)

☐ Education/training of staff and patients
☐ Switching single use items to reusable items (ex. plastic garment bags, surgical gowns/caps)
☐ Increasing exposure to nature (ex. Nature for Healing)
☐ Optimizing drugs and devices (ex. switching to low carbon anaesthetic gases)
☐ Optimizing food sourcing (ex. improving patient provided food sourcing, plant-based foods)
☐ Reducing the amount of unused surgical instruments (i.e., less unnecessary sterilization)
☐ Reducing the amount of energy used (i.e., lighting, heating/cooling)
☐ Increasing leadership to create a culture of sustainability and meet goals
☐ Better labelling of products and waste bins

Are there any opportunities for improvement not mentioned above that you can identify?

BARRIERS

Which of the following do you believe are barriers to improving the environmental sustainability in the hospital? (Please select all that apply)

☐ Cost
☐ Lack of support
☐ Lack of education
☐ Lack of resources
☐ Negative attitudes
☐ Lack of time
☐ Lack of incentive
☐ Inconvenience

GENERAL KNOWLEDGE/ATTITUDES/PERCEPTIONS ON CLIMATE CHANGE

Please state the level of agreement with the following statements:

	Strongly disagree	Somewhat disagree	Neutral	Somewhat agree	Strongly agree
In general, environmental sustainability is important to me	○	○	○	○	○
I consider the environment when making decisions in my daily life	○	○	○	○	○
I believe that improving environmental sustainability should be a priority	○	○	○	○	○
I would like to get involved in initiatives that help improve environmental sustainability	○	○	○	○	○

Any other comments you would like to add?
